# Patient Satisfaction With Dental Services

**DOI:** 10.7759/cureus.49223

**Published:** 2023-11-22

**Authors:** Eman J Al Ghanem, Nora A AlGhanem, Zahra S AlFaraj, Layla Y AlShayib, Dhuha A AlGhanem, Walla S AlQudaihi, Sara Z AlGhanem

**Affiliations:** 1 Dental Hygiene, North of Riyadh Dental Clinic, Riyadh, SAU; 2 Dentistry, Redah Healthcare Center, Qatif, SAU; 3 Dentistry, North of Riyadh Dental Clinic, Riyadh, SAU; 4 General Medicine, Dammam Medical Complex, Dammam, SAU; 5 Dentistry, Huraymala General Hospital, Riyadh, SAU; 6 Dentistry, Al Nasserah Primary Healthcare Center, Qatif, SAU

**Keywords:** service, rural, dental, satisfaction, patient

## Abstract

Patient satisfaction is an integral aspect of healthcare quality assessment, and it plays a crucial role in evaluating the effectiveness of healthcare services. This systematic review investigates patient satisfaction with dental services provided by public dental hospitals in rural and remote areas of Saudi Arabia. The study conducted a systematic review following the Preferred Reporting Items for Systematic Reviews and Meta-analysis (PRISMA) standards. It involved a comprehensive search across multiple databases, including Medline, Cochrane, Embase, and CINAHL, with tailored search strategies for each database using MeSH terms and keywords. To ensure inclusivity, the search covered publications in both English and Arabic and included Google Scholar for gray literature. Inclusion criteria focused on empirical studies conducted in rural and remote public hospitals in Saudi Arabia, published between 2013 and January 2023, assessing patient satisfaction in oral or dental care for adult patients. Data screening and extraction followed a rigorous two-step process, and a narrative synthesis was used to analyze and summarize the findings. The findings reveal a complex landscape of patient satisfaction in these settings, with varying levels of contentment reported. While more than 50% of patients expressed satisfaction with the quality of dental care, significant challenges related to accessibility were evident. Patients residing in remote and rural areas often had to travel long distances to access dental clinics, resulting in dissatisfaction. Demographic factors, particularly education and age, were identified as significant influencers of patient satisfaction, with more educated individuals tending to be less satisfied. This study emphasizes the importance of continuous monitoring of patient satisfaction to enhance service delivery, particularly in public dental clinics serving remote and rural areas. Addressing issues related to access, availability, clinical quality, and effective communication is vital for improving patient satisfaction in these healthcare settings. The study concludes with recommendations for policymakers, including the development of quality assurance policies, cost mitigation strategies, and targeted interventions to address demographic disparities and enhance patient satisfaction with dental care services.

## Introduction and background

The majority of healthcare research is focused on quality assurance, or the continuous improvement of quality aspects of service standards [1]. Today, there is widespread agreement that it is impossible to provide high-quality health care without measuring patient satisfaction. As a result, in many countries, patient satisfaction is recognized as an important aspect and critical indicator of the quality of dental care provided [2]. Researchers have agreed that when the care provided exceeds the patient's expectations, the quality of healthcare services is high. However, satisfaction will differ depending on the individual's perception of quality [3]. Various studies have highlighted the significance of patient satisfaction. Patient satisfaction plays a vital role in evaluating the quality of healthcare. It integrates a consumer perspective into healthcare policy decisions and respects patients' rights and opinions [1]. Satisfied patients are more inclined to adhere to healthcare professionals' treatment recommendations, revisit the same healthcare facility when necessary, and recommend it to others seeking care [1]. Consequently, patient satisfaction serves as a valuable metric for assessing the quality of care, bringing a consumer viewpoint into healthcare policy considerations, and valuing patients' rights and perspectives; hence, it holds a crucial role in improving any healthcare system [2].

In dental care, just like in other healthcare services, patient satisfaction can be used to determine the strengths and weaknesses of the dental services and, hence, help to raise the quality of treatment and future planning. Moreover, the evaluation of the satisfaction of patients reflects the existing disparities in healthcare, especially in the context of remote and rural communities [3]. In addition to that, the degree of agreement between the patient's preexisting expectations and impressions of the actual care received has an impact on patient satisfaction [4]. Some of the main components of patient satisfaction include quality, affordability, accessibility, patient characteristics, and general satisfaction. Patient satisfaction regarding the quality of services is one of the most essential subjects globally. In Saudi Arabia, the government is investing to ensure that quality care is offered.

Quality is often defined as being suitable for its intended purpose, a concept often referred to as “fitness of use” [5]. When it comes to services, the quality of a service can be closely tied to the satisfaction levels of the customers who receive it. In healthcare, patient satisfaction serves as an important metric for evaluating and tracking the quality of services delivered by hospitals. It offers insight into the overall quality of healthcare institutions. Therefore, measuring satisfaction is an essential tool for purposes like administration, research, and planning in the healthcare sector. Another factor, accessibility to healthcare services, entails the time required to receive services, physical facilities, working hours, and service processes. There are three main dimensions to service accessibility: the physical dimension (cost and waiting time), economic accessibility, and information (informed choice regarding quality and type of service) [4]. Evidence shows that the most relevant aspects when it comes to choosing a dentist include convenience and accessibility, and patients will prefer dental hospitals located near their homes and easily accessible [6]. Several studies conducted to determine different levels of satisfaction with care given in different sectors have identified affordability as one of the most important factors. A study from 2013 showed that the selection of a dental clinic is mainly based on affordability, convenience, and accessibility [7].

One of the most frequently cited reasons for seeking treatment from public hospitals was the affordability of the services [5,8]. In addition to these factors, patient characteristics are essential determinants on the patient satisfaction scale, broadly including education, age, and gender. Higher-educated people have higher expectations of the quality of services and are hence less satisfied compared to those with low education [5]. With regards to gender, males and females have varying psychological and physical status, and hence, it is assumed that different genders will have different feelings about different services [9].

Keeping the different aspects of quality assurance and patient satisfaction in view, this study sought to determine the levels of patient satisfaction with the quality of dental care among services offered by public dental care institutions in Saudi Arabia's rural and remote settings. Despite the fact that many health strategic plans are being developed by Saudi Arabian policymakers for remote and rural areas, more research on the quality of dental healthcare will support the planning of dental health programs. Moreover, there is a lack of systematic reviews on patient satisfaction in rural and remote settings in Saudi Arabia. Filling this research gap is important for a comprehensive understanding of the challenges and opportunities in these areas. Policymakers can also use data on patient satisfaction to formulate policies that prioritize patient-centered care, thereby aligning healthcare services with the needs and preferences of the population.

## Review

Methodology 

The current study was done as a systematic review following the Preferred Reporting Items for Systematic Reviews and Meta-analysis (PRISMA) standards.

Search strategy

A systematic search was conducted to identify various research studies addressing patient satisfaction with dental services in different regions of Saudi Arabia. Multiple databases, including Medline, Cochrane, Embase, and CINAHL, were scrutinized to retrieve these studies. The search strategy was tailored for each database, considering subject headings and search structures. Both MeSH terms and text keywords were employed, ensuring relevance to the study's focus. To maximize sensitivity and encompass diverse publications in different languages, papers published in both English and Arabic were examined. In addition, Google Scholar was utilized to search for gray literature using relevant subject keywords. Furthermore, the search methodology for all databases was refined, incorporating suitable syntax, subject headings, and controlled vocabulary to enhance the search results' sensitivity. The study utilized a range of search terms, including "remote," "public dental clinics," "dental services," "dental clinics," "dental healthcare," "rural," "patient satisfaction," "patient attitude," "Saudi Arabia," and "patient dissatisfaction." These terms were employed in various combinations to generate a wide array of research papers.

Inclusion and exclusion criteria

The study incorporated multiple empirical investigations that specifically addressed patient satisfaction with dental services in rural and remote regions of Saudi Arabia. The selected articles and reports had undergone peer review and were published between 2013 and January 2023. The choice to focus on studies conducted within the last decade was deliberate, given the rapid transformation occurring in both the Saudi Arabian and global healthcare sectors. This recent timeframe was deemed essential to provide up-to-date insights into patient perspectives on dental services, aligning with Saudi Arabia's substantial investments in healthcare sector improvements. The inclusion criteria for the studies were as follows: they had to be conducted in remote and rural public hospitals in Saudi Arabia or contain elements related to rural areas. Furthermore, these studies needed to assess patient satisfaction specifically in the context of oral or dental care. They also had to be published in either English or Arabic and involve adult patients who had received dental care within the past year. Research studies that reviewed healthcare records reporting on patient satisfaction with oral care were also considered in the analysis. Primarily, the articles included needed to be pertinent to the research questions and topic under investigation and grounded in robust scientific methodologies. Additionally, they were expected to demonstrate reliability and objectivity in their findings, which could be assessed by the transparency of their data collection methods. On the other hand, the study excluded various types of research, such as those conducted in urban areas or private hospitals. Studies assessing different aspects of dental care, those exclusively involving pediatric patients, research conducted before 2013, and non-empirical studies like editorials, case reports, and letters were also excluded from the research analysis.

Data screening and extraction

The results obtained from the search and the identified articles were imported into the EndNote software. To eliminate any duplicate research studies, a comparison was made by examining the titles, authors, and publication details of the articles. The researcher employed a two-step methodology to assess the relevance of articles with respect to the research question. Initially, a review of titles and abstracts was conducted to determine their relevance. Any discrepancies were resolved through consultation with the researcher. Subsequently, a comprehensive assessment of the full texts of studies that were considered relevant in the first stage was carried out using established eligibility criteria. A standardized data extraction form was employed to extract information from the included articles. This form included details about the study characteristics (authors, publication year, study design, sample size), participant characteristics (socioeconomic status), and primary outcomes (patient satisfaction scores and satisfaction with various aspects of care). The extracted data were organized into a summary table for comparison. A narrative synthesis was undertaken to analyze the findings from the included studies. Both textual descriptions and tables were employed to condense and elucidate the key characteristics and outcomes of these studies. Variables extracted from the research studies encompassed details such as the author and publication year, study objectives, research design, sample size, as well as the findings and conclusions derived from each study.

Results

**Figure 1:**
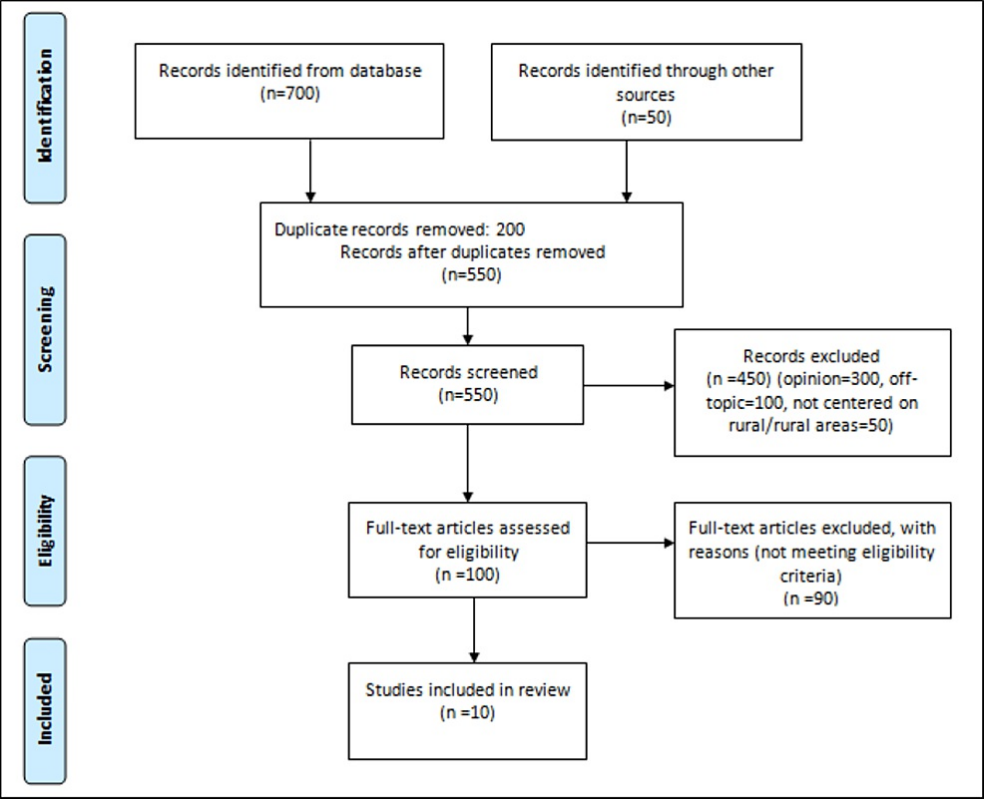
PRISMA diagram

The PRISMA flowchart diagram (Figure 1) illustrates the search outcomes derived from various electronic databases used in the data retrieval process, outlining the selection process and the reasons for exclusion. Initially, approximately 700 articles were reviewed, with an additional 50 sourced from other outlets. A check for duplicates resulted in the removal of 200 articles. Subsequently, the titles and abstracts of the remaining 550 articles were evaluated, leading to the exclusion of 450 studies that did not align with the study criteria. In the final phase, a comprehensive review of the full text was conducted for the remaining 100 articles, ultimately culminating in the inclusion and summarization of nine studies within the research review.

Characteristics of the studies

A total of nine research studies [10-18] were carefully examined, adhering to specific inclusion and exclusion criteria. These studies were published over a decade, spanning from 2012 to 2022. The research encompassed diverse methodologies, including cross-sectional surveys, observational studies, and reviews, with varying sample sizes. The sample sizes of these studies ranged between 150 and 500 patients. These studies provided valuable insights into patient satisfaction with dental services, shedding light on a range of factors that influence this critical aspect of healthcare. These studies collectively provide a multifaceted view of patient satisfaction with dental services in Saudi Arabia, touching on factors like access, sociodemographic characteristics, waiting times, and overall service quality. The studies utilized diverse objectives, study designs, and sample sizes to explore patient satisfaction comprehensively. Geographically, the studies encompassed various regions within Saudi Arabia, ranging from Hail to Jazan, offering a comprehensive perspective that included both urban and rural settings.

Outcome measures

The findings of these studies presented a diverse range of responses from patients who sought dental care services. While some of the studies reported a notably higher level of patient satisfaction, others shed light on significant shortcomings in terms of the quality and accessibility of dental clinics or hospitals, ultimately resulting in patient dissatisfaction. An overarching consensus among all these studies was the recognition of a considerable gap in the existing body of evidence related to this specific area of research. These findings underlined the intricate nature of patient satisfaction in dental care and emphasized the need for further research to address disparities, gain deeper insights into patient perspectives, and enhance dental service delivery in Saudi Arabia. To provide a clearer understanding of these studies, a compiled summary of their primary characteristics is illustrated in Table 1.

**Table 1:** Baseline characteristics and findings of the included studies

Author	Objectives	Study design	Sample size	Findings	Conclusion
Siddiqui et al. (2017) [10]	Evaluate the level of patient satisfaction with treatment expectations and dentist behavior.	Cross-sectional design conducted in Hail rural areas.	153	It was observed that the expectations of patients were below the satisfaction level in most parameters.	The analysis results showed a satisfaction level of 88.9%, but further research studies were further recommended.
Tashkandi, F. S et al. (2017) [11]	To investigate the level of patient satisfaction with services and dental care provided and discover the weaknesses and strengths of the services in Saudi Arabian rural areas.	Questionnaire-based cross-sectional analytical observational study.	150	The majority of patients were satisfied with the four main aspects tested (appointments, facilities, staff, and treatment). Overall, patient satisfaction has a prevalence rate of 88.5%. Patients were satisfied with the communication, professionalism, and explanations given. The main reason for dissatisfaction was the long waiting time before the appointment.	The majority of patients were satisfied with the dental care services given.
Orfali, S., et al. (2020) [12]	To determine the disparity in the use of dental services in Saudi Arabia in various regions and on related factors.	Review.	--	Dental service use was lower among people living in rural areas due to factors such as physical access and limited availability. Hence, the level of dissatisfaction was high.	High dissatisfaction level due to inequalities. More studies are needed to evaluate factors associated with inequality and whether they have been increasing or decreasing in Saudi Arabia.
Quadri F. A., et al. (2018) [13]	To know the factors that play a role in influencing dental service utilization in Jazan rural areas, Saudi Arabia.	A cross-sectional survey.	395	The research findings portrayed past bad experiences with oral healthcare providers.	The study concludes that even though Saudi Arabia provides free primary oral health services, many barriers exist, especially in rural areas.
Shubayr M. A., et al. (2022) [14]	To assess the geographic accessibility of dental clinics located in public primary healthcare clinics (PHCs) and hospitals in the Jazan region of Saudi Arabia.	Observational study.	--	There is a major accessibility issue, as many people require extensive travel time to access healthcare.	The study shows that there are accessibility issues in Jazan whereby Jazan residents have to travel long distances to access dental care in public hospitals.
Khan A. A., et al. (2018) [15]	To investigate the effect of multiple sociodemographic characteristics on patient satisfaction levels in dental practices in Saudi Arabia.	Questionnaire-based survey.	500	Satisfaction levels differed significantly by education level (P< 0.001) and the type of clinic (P<0.001). The satisfaction score was higher for private clinics compared to public clinics.	The study revealed the effect of sociodemographic variables on overall satisfaction scores, with rural and remote public hospitals having low patient satisfaction levels compared to the private sector.
Balhaddad A. A. et al. (2018) [16]	To evaluate patient satisfaction and the associated factors with services, facilities and treatment in dental care in Saudi Arabia.	Cross-sectional Study.	262	More educated patients are less satisfied, while less educated patients are more satisfied.	Most of the patients were satisfied with the quality of dental services, and education and age were significantly associated with their satisfaction.
Saleh Al Ateeq et al. (2022) [17]	To assess patient satisfaction with healthcare services in the dental clinics in Saudi Arabia using PSQ-18.	Cross-sectional study.	300	54.3% were satisfied with the service provided by the dental clinics.	The study showed that slightly more than half of the patients in dental clinics were satisfied with health services.
Mahrous M. S., et al. (2012) [18]	To determine patients’ satisfaction regarding the quality of dental care at dental clinics	Cross-sectional analytical observational study.	202	The ranking of factors related to patient satisfaction revealed a percentage of 79.5% agreement for the disciplines of patient satisfaction, which showed high satisfaction levels.	The majority of patients were satisfied with the location, efficiency, and technical competency.

Discussion 

This study assessed the satisfaction for dental services among the rural population, which is of utmost importance since the research in recent times has been more focused on urban settings; unfortunately, this cohort is mostly ignored, which is possibly a reason for healthcare disparities. Therefore, to achieve the concept of universal health equity, or health for all, consideration of this population is highly important. The findings of our study are significant to report in this regard since we specifically targeted the rural population, and in addition to evaluating the satisfaction levels, our findings demonstrated that multiple factors are responsible for or contribute to achieving ideal levels of patient satisfaction with dental services, specific to Saudi Arabia.

Demographic factors

Among our included studies, education and age were found to be significant demographic factors affecting patient satisfaction. Patients with lower levels of education tended to report higher satisfaction [15,16]. This may suggest that patients with higher education levels have higher expectations. Similarly, Alhozgi et al. described that patients' age, education, gender, marital status, location of residence, income, and race are among the demographic and socioeconomic factors that are thought to be major drivers of their level of satisfaction with their healthcare system [19]. Additionally, Shelke et al. stated that the utilization of dental care in community health centers in rural locations is decreased by adverse sociodemographic variables such as older age, absence of insurance, poorer income, and ethnic views on health care [20].

Accessibility

In our review, accessing dental care services in rural and remote regions presented significant challenges. Patients often had to undertake long-distance travels, sometimes spanning hours, to reach the nearest dental facility [13,14]. The significance of this theme is underscored by the findings of reviewed studies, which demonstrate that patients residing in such remote areas encounter various hurdles when attempting to access dental care, primarily attributable to extended travel times [12]. Furthermore, another study highlighted the substantial accessibility challenge, with many individuals compelled to invest significant time and effort in accessing healthcare [14]. Lee et al. narrated that in remote and rural regions, there is nevertheless a persistent mix of insufficient availability and access to timely oral health care. The quality of life of those residing in rural locations may be significantly reduced if they do not have access to possibilities for maintaining dental health [21]. While Barnett et al. described that rural and remote individuals may attend mobile dental facilities to receive care if there is not a dentist in their area, this can be challenging because of the erratic and unpredictable nature of the services. They might also go to a dentist in a different town, but depending on their insurance coverage and the length of the trip, this can frequently result in the patient and their family having to pay more [22]. Similarly, in our study, rural residents in Jazan were also reported to frequently face extended travel distances to access dental care services in public hospitals. As such, key determinants of access include the geographic distribution and number of available dental clinics, along with transportation options. Addressing this issue is crucial for enhancing access to dental care in remote areas and ultimately promoting higher levels of patient satisfaction.

Quality of dental care

Given the influence that quality has on the recipients of services, quality and quality management, which continues to be one of the most significant challenges in the dental field, have become increasingly contentious topics. But despite these difficulties, quality management plays an important role in guaranteeing the provision of high-quality dental care services [23]. This aspect is of utmost importance, as patients worldwide typically expect healthcare providers to possess the necessary skills and knowledge for diagnosing and treating their dental issues. In this study, the influence of predisposing factors and enabling resources on patients' perceptions of the quality of dental services they received was established [13,16]. Many patients expressed satisfaction with the quality of dental care services, including the facilities. However, they also recommended increased investment in facilities and infrastructure to enhance the accuracy of diagnoses and safety procedures [10]. Notably, in several studies included in our review, a substantial proportion of patients (more than 50%) reported satisfaction with the quality of dental care they received [12-14]. Similarly, the results of a study by Bansal et al. reported that the majority of patients expressed satisfaction with the caliber of care they received; however, some patients expressed dissatisfaction or felt compelled to file complaints. Therefore, the authors suggested that a dentist should not only focus on completing the procedure as quickly as possible but also on fully explaining the procedure and the available options to the patient [24]. Moreover, Moshkelgosha et al. narrated that the standard of dental care received has a significant impact on the outcome of the procedure. Based on earlier research, it was anticipated that patient satisfaction would be essential to drawing new patients to a dental facility. Patients typically look for high-quality dental care services performed by competent dentists using modern technologies and also advocate these services to their friends and family [25].

Communication

Numerous research studies have demonstrated the significance that patients place on the quality of the relationship they have with their dentist. Furthermore, the perception of patient satisfaction as a crucial component in evaluating the caliber of oral healthcare is growing. Nevertheless, patient satisfaction is a complicated construct made up of both objective and subjective components. Communication is a key component of this connection, and research has repeatedly shown a strong correlation between patient satisfaction and the caliber of communication between the dentist and the patient. According to certain theories, feeling satisfied with a dentist can help reduce stress, which could then lead to even more contentment and satisfaction. Numerous studies have emphasized the significance that patients place on dentists' openness to talking about their perceived discomfort and fear [26]. Similarly, in the studies we reviewed, Siddiqui et al. [10] observed that patients expressed satisfaction with the communication process, noting the professionalism and clarity of explanations provided by dental care providers. Moreover, the importance of offering clear explanations to patients was also noted to be an important factor, although it was concluded that patient age can influence how they perceive this aspect. Enhancing communication plays a crucial role in elevating patient satisfaction levels and alleviating anxiety or fear associated with dental procedures [10,13].

Waiting time

Most often, patients' interactions with hospitals start when they are waiting in an outpatient department. Thus, waiting times are thought to be among the key markers of high-quality services. Extended wait times are a prevalent issue since there is a greater demand than there is a supply of medical resources. Extended waiting periods may exacerbate medical conditions in patients, erode public trust in healthcare institutions, and lower patient satisfaction [27]. In our study, among patients who reported dissatisfaction, the primary reason identified was the prolonged waiting period before their appointments [11]. This observation aligns with findings from other studies, which emphasized that waiting is a prevalent issue contributing to patient dissatisfaction [28]. Furthermore, additional factors contributing to waiting times included the duration required for each dental care procedure and the efficiency of dental clinic operations [17,18]. Addressing and reducing wait times can play a pivotal role in enhancing patient satisfaction and optimizing clinic efficiency. The literature reviewed consistently highlighted a significant association between patient satisfaction and waiting times in dental clinics, underscoring the pivotal role of waiting times in shaping patient satisfaction [28]. Similarly, the results of the study by Inglehart et al. reported that those who received care from early arriving doctors reported higher levels of satisfaction, were more likely to plan to follow their provider's advice, and had a more favorable relationship with their provider than those in the late-arriving group, who responded to all questions negatively. Prolonged wait times before a dental appointment negatively impact patients' satisfaction levels, their opinions of the relationship between the patient and the provider, and their likelihood of returning [29].

Cost

The theme of the cost of dental care services is crucial, as it is widely recognized as a significant barrier to accessing dental care. The elevated cost of care was primarily associated with factors such as extended travel times and accessibility challenges, particularly the need for extensive travel to access healthcare [14]. Mitigating the cost of dental care can play a pivotal role in enhancing patient satisfaction and ensuring that patients have access to the necessary care. Affordability and reasonable fees were identified as factors that encourage patient satisfaction [8]. However, it's essential to acknowledge that this study primarily focused on schools situated near public hospitals offering dental services. Results of a survey showed that, when compared to participants who felt their oral health was good, those who rated their oral health as poor found the high cost of dental care to be the most discouraging issue (P = 0.05) [8]. Results of another survey concluded that, compared to other aspects of healthcare quality, patients' satisfaction with the financial accessibility of dental care was shown to be lower [30].

A wide range of complex factors influence the utilization of dental care. Individuals' decisions to seek professional dental aid or forego care are influenced by a variety of factors, including behavioral, socioeconomic, and culturally associated predisposing enabling factors and need-based factors. A sufficient understanding of how people use health services and the variables that influence this behavior is necessary to enhance oral health outcomes [31]. Patient satisfaction has the potential to improve treatment quality and future planning in dentistry practice by identifying the strengths and weaknesses of dental clinics. Since patient satisfaction is a multifaceted concept that takes into account several factors, such as expectations, lifestyle choices, medical history, and educational background, it may be more appropriate to measure different aspects of dental health care. Thus, in the field of dentistry, patient satisfaction can be used to identify the advantages and disadvantages of dental treatments as well as to improve the standard of care [28]. Due to factors such as a lack of dental professionals, geographic isolation, inadequate infrastructure, and lower socioeconomic standing, people who live in rural and isolated locations often struggle with oral health issues and have difficulty accessing care. When addressing health disparities, it is important to consider that a global measure of satisfaction with delivered care may reflect multiple aspects of care, particularly in the context of rural and distant settings [32].

Recommendations

On the basis of our findings, we recommend that the government should develop and implement quality assurance policies tailored to rural areas to improve the quality of dental services and patient satisfaction. Ensuring that all dental clinics are well-equipped is essential for building trust in the care provided. Additionally, addressing the cost burden on rural and remote area patients by introducing alternative payment methods, such as insurance for dental services, can make dental care more affordable and accessible to a broader population. Establishing a dedicated quality control unit within the healthcare department to enforce quality assurance policies is also crucial. Regular evaluations of patient satisfaction in rural areas should be conducted to ensure that the quality-of-care matches that in urban areas. Moreover, steps should be taken to increase the availability of dental clinics in rural and remote areas. In this regard, mobile dental clinics can also be a great initiative to reduce accessibility issues in rural pockets of Saudi Arabia. In addition to that, the development of targeted interventions within public dental clinics to address disparities linked to demographic factors is essential to address disparities related to the sociodemographic characteristics of the patients. Educational programs can also be designed to educate less educated and older individuals about the expected quality of dental services, considering their limited access to care.

The systematic review has identified various factors contributing to patient satisfaction in dental care concerning rural location. However, further in-depth studies should explore the antecedent factors that lead to patient satisfaction or dissatisfaction. It is crucial to understand patients' needs and expectations, not only in terms of desired services but also their minimum acceptable service standards.

## Conclusions

In conclusion, the review of existing studies on patient satisfaction with dental care services reveals a mixed landscape of opinions. While more than half of the patients expressed satisfaction with the quality of care, significant challenges persist, especially in remote and rural areas where access to care remains a major concern. Education and age emerged as influential factors, with more educated patients tending to be less satisfied. This suggests that addressing access issues and maintaining high clinical quality are essential for enhancing patient satisfaction, particularly in public dental clinics serving underserved areas. Patient satisfaction remains a critical metric for assessing healthcare quality and should be closely monitored to drive improvements in service delivery.
